# Pathways of cell-cell transmission of HTLV-1

**DOI:** 10.3389/fmicb.2012.00378

**Published:** 2012-10-24

**Authors:** Claudine Pique, Kathryn S. Jones

**Affiliations:** ^1^CNRS UMR 8104, INSERM U567, Université Paris-Descartes, Institut CochinParis, France; ^2^Cancer and Inflammation Program, Basic Research Program, SAIC-Frederick, Inc., Center for Cancer Research, National Cancer Institute, Frederick National Laboratory for Cancer ResearchFrederick, MD, USA

**Keywords:** retrovirus, HTLV-1, cell–cell transmission, HTLV-1 infectivity, T cell, antigen-presenting cells, HTLV-2, virological synapse

## Abstract

The deltaretroviruses human T cell lymphotropic virus type 1 (HTLV-1) and human T cell lymphotropic virus type 2 (HTLV-2) have long been believed to differ from retroviruses in other genera by their mode of transmission. While other retroviruses were thought to primarily spread by producing cell-free particles that diffuse through extracellular fluids prior to binding to and infecting target cells, HTLV-1 and HTLV-2 were believed to transmit the virus solely by cell–cell interactions. This difference in transmission was believed to reflect the fact that, relative to other retroviruses, the cell-free virions produced by HTLV-infected cells are very poorly infectious. Since HTLV-1 and HTLV-2 are primarily found in T cells in the peripheral blood, spread of these viruses was believed to occur between infected and uninfected, T cells, although little was known about the cellular and viral proteins involved in this interaction. Recent studies have revealed that the method of transmission of HTLV is not unique: other retroviruses including human immunodeficiency virus (HIV) are also transmitted from cell-to-cell, and this method is dramatically more efficient than cell-free transmission. Moreover, cell–cell transmission of HTLV-1, as well as HIV, can occur following interactions between dendritic cells and T cells, as well as between T cells. Conversely, other studies have shown that cell-free HTLV-1 is not as poorly infectious as previously thought, since it is capable of infecting certain cell types. Here we summarize the recent insights about the mechanisms of cell–cell transmission of HTLV-1 and other retroviruses. We also review *in vitro* and *in vivo* studies of infection and discuss how these finding may relate to the spread of HTLV-1 between individuals.

## INTRODUCTION

Human T cell lymphotropic virus type 1 (HTLV-1) was the first pathogenic retrovirus discovered in humans (Poiesz et al., 1980). This virus is the only human retrovirus known to be the causal agent of a cancer, a neoplasia called adult T cell leukemia (ATL; Yoshida et al., 1982, 1984). HTLV-1 is also associated with several inflammatory disorders, primarily a progressive neurological disease named HTLV-1-associated myelopathy/tropical spastic paraparesis (HAM/TSP; Gessain et al., 1985; Osame et al., 1986). The closely related retrovirus human T cell leukemia virus type 2 (HTLV-2) does not cause leukemia, although some infected individuals develop mild lymphocytosis and, occasionally, neurologic symptoms (Hjelle et al., 1992; Dooneief et al., 1996; Feuer and Green, 2005; Biswas et al., 2009). In the peripheral blood of infected individuals, both HTLV-1 and HTLV-2 are primarily found in T cells, and both viruses can immortalize T cells in culture (reviewed in Feuer and Green, 2005). ATL is a malignancy of CD4^+^ T cells, and HTLV-1 primarily infects CD4^+^ T cells, while HTLV-2 primarily infects CD8^+^ T cells (Kira et al., 1991; Lal et al., 1995; Wu et al., 1996; Manns et al., 1999; Nagai et al., 2001; Murphy et al., 2004). Dendritic cells (DCs), B cells, and monocytes can also be infected in individuals with HTLV (Macatonia et al., 1992; Koyanagi et al., 1993).

Like other retroviruses, HTLV-1 and HTLV-2 enter target cells following specific interactions between the viral envelope glycoprotein (Env) and cellular receptors. Efficient entry of HTLV-1 has been shown to involve three distinct molecules: heparan sulfate proteoglycans (HSPGs) and Neuropilin 1 (NRP-1) for the initial binding to the cell, and glucose transporter 1 (GLUT1) for entry (Manel et al., 2003; Jones et al., 2005; Ghez et al., 2006; Lambert et al., 2009). HTLV-2 binding and entry also involves NRP-1 and GLUT1, but differs from HTLV-1 in that it does not require HSPG. The studies identifying the HTLV receptors have recently been extensively reviewed (Ghez et al., 2010; Ilinskaya et al., 2010; Jones et al., 2011; Hoshino, 2012) and thus will not be detailed here.

For many years, HTLV-1 and HTLV-2 were believed to differ from retroviruses in other genera in their mode of transmission. For the other retroviruses, viral spread was thought to require the production of cell-free viral particles that diffuse through extracellular fluids and subsequently bind to, enter, and infect target cells. For HTLV-1 and HTLV-2, it was believed that the cell-free virus was poorly infectious, and that the viruses could only be efficiently spread by direct contact between infected cells and target cells (cell–cell transmission).

The belief that HTLV-1 is poorly infectious as a cell-free particle originated soon after its discovery and was based on both *in vivo *and *in vitro* observations. Studies of transfusion suggested that cell–cell contact is required for HTLV-1 transmission: although a high percentage of individuals receiving cellular blood components (whole blood, red blood cells, or platelets) from HTLV-1- or HTLV-2-infected individuals become infected with the virus, the recipients of non-cellular blood products (plasma fraction or plasma derivatives) from infected individuals do not become infected (Maeda et al., 1984; Miyamoto et al., 1984; Jason et al., 1985; Lairmore et al., 1989). In one study directly comparing transmission following transfusion of plasma from individuals with different human retroviruses, seroconversion occurred in 89% of the individuals who received plasma from HIV-1 infected individuals, but in none of the individuals who received plasma from individuals with HTLV-1 or HTLV-2 (Donegan et al., 1994).

*In vitro* experiments supported the notion that the cell-free virus is poorly infectious. Although in the peripheral blood the virus is primarily found in T cells, early studies showed that cell-free HTLV-1 and HTLV-2 do not efficiently infect or transform primary T cells isolated from the peripheral blood *in vitro*. In contrast, primary T cells can become infected and transformed following coculture with either HTLV-1-infected or HTLV-2-infected cell lines (Yamamoto et al., 1982; Popovic et al., 1983; Green and Chen, 1990).

However, other *in vitro* studies showed that cell-free HTLV-1 is not completely non-infectious. Early studies reported rare infection of T cells (de Rossi et al., 1985) and non-lymphoid cells (Clapham et al., 1983) by cell-free virus. Later studies using more sensitive assays reported that a number of T and B cell lines (Fan et al., 1992; Agadjanyan et al., 1994; Jinno et al., 1999), as well as cell lines of non-lymphoid origin (Graziani et al., 1993; Haraguchi et al., 1994), could be infected following exposure to cell-free virus, although at a very low level. More recent studies with DCs have confirmed and extended the notion that cell-free HTLV-1 can be infectious. Several groups have demonstrated that the primary DCs, unlike T cells, are routinely infected after exposure to cell-free virus (Jones et al., 2008; Jain et al., 2009; Lambert et al., 2009; Valeri et al., 2010)*. *In addition, in contrast to what occurs in cultures of purified T cells, T cells cocultured with DCs routinely become infected after the addition of cell-free virus. Further studies showed that when DCs are exposed to cell-free virus, they rapidly transmit the virus to T cells. These observations suggest that there may be blocks to infection after the cell-free particle binds to T cells that are not present when the virus is presented by DC cells to T cells, or that interacting with the DCs alters the virus in a way that allows it to infect T cells.

As described above, several early studies showed that the plasma from HTLV-1- and HTLV-2-infected individuals does not infect recipients of transfusions. Originally, these studies were interpreted as reflecting the fact that cell-free HTLV-1 and HTLV-2 virions in the blood are far less infectious and/or more labile than HIV-1 virions. However, later studies revealed that cell-free viral particles are only rarely be detected in plasma or serum from HTLV-1- or HTLV-2-infected individuals. Other studies revealed that, even in individuals with a high percentage of PBMCs containing integrated HTLV-1 genomic DNA, little or no viral mRNAs or proteins are detected in PBMC immediately after isolation (Gessain et al., 1991; Richardson et al., 1997; Moritoyo et al., 1999). These observations indicate that the lack of infection through acellular blood products is due to the lack of detectable levels of virus in peripheral blood, and thus does not support the notion that HTLV-1 particles are inherently non-infectious.

The lack of viremia in individuals with HTLV-1 is in sharp contrast to the high levels of viremia and active replication found in HIV-1-infected individuals. Indeed, the method of persistence of HTLV-1 in infected individuals appears to be very different from that of HIV-1. The peripheral blood of HTLV-1-infected individuals contains clones of large numbers of infected cells with the same integration site (Wattel et al., 1995; Leclercq et al., 1998; Zane et al., 2009), indicating that they are derived from a single infected cell. Further studies revealed that specific clones can persist over years in a given individual (Cavrois et al., 1996; Etoh et al., 1997; Cavrois et al., 1998). A recent characterization of infected cells using high-throughput methods revealed that, in a typical HTLV-1-infected individual without ATL, there are between 500 and 5000 clones and the majority of these clones are maintained over a period of years (Gillet et al., 2011).

The presence of these clones indicates that, rather than spreading from cell-to-cell, HTLV-1 persists in individuals primarily by mitotic replication of infected cells*.* Consistent with this, the percentage of infected cells (referred to as the HTLV-1 proviral load) remains stable within an individual over time. Moreover, unlike HIV-1, the HTLV-1 genome shows very little variation within an individual, consistent with it being replicated by cellular DNA polymerase during division of infected cells rather than the more error-prone reverse transcriptase. Taken together, these observations have lead to the belief that HTLV-1 persists in two stages in an individual. Soon after an individual is exposed to the virus, HTLV-1 spreads from cell-to-cell. Later, during the chronic stage of infection, the virus persists via clonal expansion, through replication of the provirus integrated into the host cell genome during the division of infected cells.

Ten years ago, little was known about the mechanism of the cell–cell transmission of HTLV-1. Since that time, imaging studies along with *in vitro* studies of infection have provided insight into the interactions between cells required for infection of T cells by HTLV-1. During this time it has also become clear that cell–cell transmission is not unique to deltaretroviruses: both HIV and the gammaretrovirus murine leukemia viruses (MLV) can also be transmitted by cell–cell contacts, and this mode of transmission is more efficient than cell-free virus. Here, we review what has recently been learned about transmission of HTLV-1, including observations that cell–cell transmission can occur between DC and T cells, as well as between T cells. We also review what has been learned about the precise interactions between cells required for the infection of the target cells by HTLV-1 and by other retroviruses during cell–cell transmission, and discuss how these finding may relate to the spread of HTLV-1 between individuals.

## THE VIROLOGICAL SYNAPSE

**FIGURE 1:**
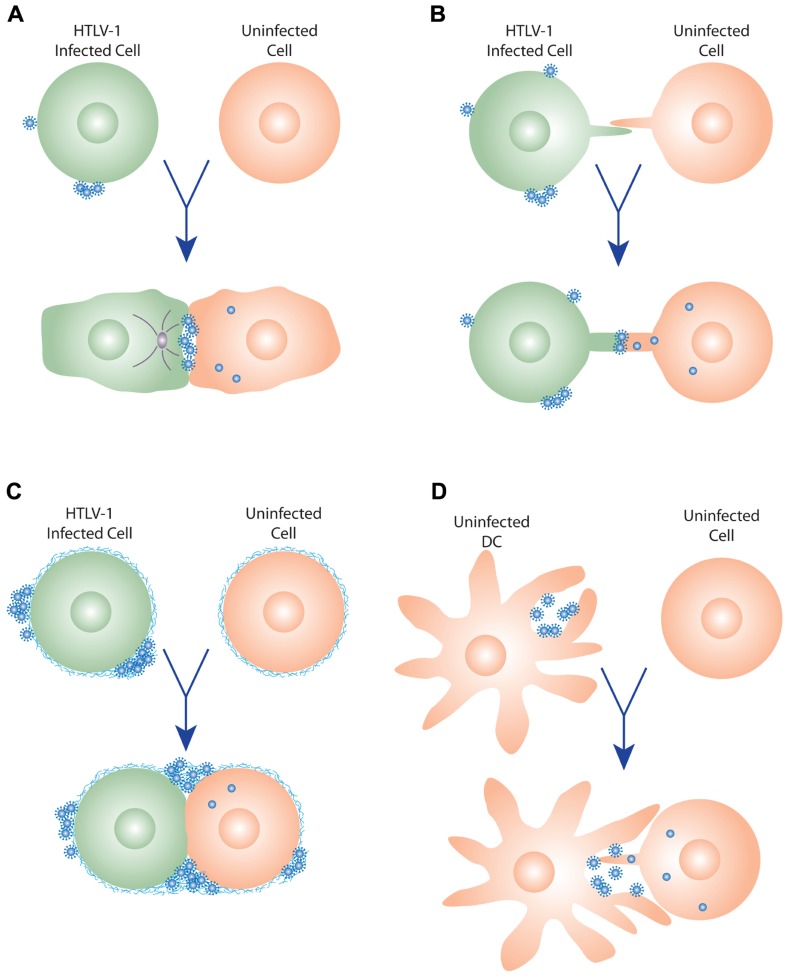
**Models of HTLV-1 cell–cell transmission**. **(A)** Virological synapse. When HTLV-1-infected cells contact uninfected cells, the virological synapse (VS) is formed by specific interactions between proteins on the infected and uninfected cells, and the MTOC (gray circle) is reoriented toward VS. Viral budding is polarized to the virological synapse, and the newly budded viruses enter the synaptic cleft formed by the VS, and then enter the target cells following interactions with the cellular receptors. (**B)** Cellular conduits. HTLV-1-infected cells can also transfer virus to uninfected cells by transient membrane extensions (cellular conduit). Interactions can occur between conduits on the infected and uninfected cell, or between cells and conduits (not shown), and particles have been observed at these contact points, suggesting that these may be a variant of the VS. **(C)** Extracellular viral assemblies. Following budding from the infected cell, the HTLV-1 virus remains associated with the cell within a matrix containing components of the extracellular matrix (ECM). Upon contact, these adhesive viral assemblies are rapidly transferred to an uninfected cell, which they subsequently infect. **(D)** Trans-infection via dendritic cells (DCs). DCs capture cell-free HTLV-1, transiently store the virus in surface-accessible compartments, and then, prior to becoming infected themselves, transfer the virus to uninfected T cells. This interaction may involve membrane extensions of the DCs and/or the T cells, as has been observed for HIV.

Although the observation that cell–cell contact is important for HTLV-1 transmission was made soon after the discovery of the virus, for many years little was known about the mechanism of cell-to-cell spread of this virus at the cellular level. In 2003, imaging techniques allowed the initial characterization of a specific type of cell contact that allows virions from an HTLV-1-infected T lymphocyte to be transmitted to an uninfected target cell (Igakura et al., 2003; **Figure 1A**). Confocal microscopy studies of T cells cultured *ex vivo* from HTLV-1-infected individuals showed that infected T cells spontaneously form conjugates with uninfected T cells. When this occurs, viral Gag and Env proteins and genomic RNAs are redirected to the point of contact between the T cells, indicating that viral assembly occurs at the junction between the infected and uninfected cells. Within 2 h after this contact, both HTLV-1 viral proteins and genomic RNAs were observed in the uninfected cell, suggesting that the virus had been transmitted to the target cell (Igakura et al., 2003). By analogy to other previously described specialized junctions, such as neuronal and immunological synapses, the structure at the junction between the infected and uninfected cell was named the virological synapse (VS; Igakura et al., 2003).

Soon after the description of the VS in HTLV-1 transmission, it was reported that HIV can also be transmitted from an uninfected to infected T cell via a VS (Jolly et al., 2004) and, as detailed below, a number of subsequent studies have more fully characterized the HIV VS. Another retrovirus, MLV, has also been shown to form a VS between infected and uninfected cells (Sherer et al., 2007; Jin et al., 2009).

For both these viruses, interactions between the virally encoded Env proteins and entry receptors are critical for the initial stage of infection, during which stable interactions are formed between the cells. For HIV, formation of the VS has been shown to be initiated by interactions between the surface (SU) envelope glycoprotein gp120 on the infected T cell and the receptor molecule CD4 on the target T cell (Jolly et al., 2004; Felts et al., 2010). Similarly, MLV infection was observed to involve interactions between its SU (gp70) on fibroblasts expressing viral proteins and the MLV receptor mCAT-1 on the target cell (Jin et al., 2009). Although it has not yet been directly examined, there is some evidence that supports the notion that Env/receptors interactions are also required to trigger the formation of the HTLV-1 VS. It has been shown that NRP-1 and Glut1 colocalize at the junction formed between an HTLV-1-infected T cell and a non-infected target T cell (Ghez et al., 2006). Moreover, blocking interactions with HSPGs or NRP-1 on T cells has also been shown to block infection following cell–cell transmission from HTLV-1-infected DC (Jones et al., 2008).

For the HIV-1 VS, it has been reported that the initial Env/receptor interaction between the infected and uninfected cells is reinforced by interactions mediated by adhesion molecules that stabilize the junction. These include specific interactions between the integrins intercellular adhesion molecule 1 (ICAM-1) and ICAM-3 and their ligand lymphocyte function-associated antigen 1 (LFA-1; Jolly et al., 2007)*. *Interactions between ICAM-1 and LFA-1 have also been shown to be critical for formation of the HTLV-1 VS (Barnard et al., 2005).**

These stable contacts between the two cells delineate an intracellular space referred to as the synaptic cleft. Electron tomography studies revealed the presence of HTLV-1 particles in the synaptic cleft between infected and uninfected primary lymphocytes (Majorovits et al., 2008). Many of the viruses observed were touching the membrane of the infected cell, the target cell, or both, indicating that HTLV-1 is released from the polarized cell into the synaptic cleft, and is efficiently transferred to the target cell. This is similar to what has been observed for HIV and MLV during formation of their VS: Gag and Env proteins of these viruses localize to the point of cell–cell contact, and the viruses bud into the synaptic cleft (Jolly et al., 2004; Hubner et al., 2009; Jin et al., 2009).

It seems that the increased efficiency of cell–cell transmission over cell-free transmission reflects at least in part a quantitative advantage, since it allows the polarized release of large numbers of concentrated particles in close proximity to the target cells. Consistent with this notion, recent studies using labeled genomes (Del Portillo et al., 2011) and 3D video microscopy (Hubner et al., 2009) have shown that cell–cell transmission of HIV via the VS, but not exposure to cell-free virus, can result in the infection of target cells by multiple virus particles.

The observation of the redistribution of the Gag and Env proteins of HTLV and other viruses, followed by the budding of virus into the VS, suggests that the viral proteins are being actively transported to the point of contact, which would likely involve active transport by the cytoskeleton. Consistent with this, the initial studies of the HTLV-1 VS reported that talin, an actin-associated cytoskeletal protein involved in cell–cell interactions, accumulated at the point of contact between the cells (Igakura et al., 2003). These initial studies also reported that the microtubule-organizing center (MTOC) was polarized toward the VS in the HTLV-1-infected cells, but not in the target cells (Igakura et al., 2003). Disrupting formation of microtubules was shown to inhibit both the polarization of the MTOC and the transmission of the HTLV-1 particle to the uninfected cell (Nejmeddine et al., 2005). Later infectivity studies using HTLV-1-based single-cycle vectors to quantify transmission between cells supported the notion that the cytoskeleton plays an important role in cell–cell transmission: disrupting either actin function or microtubule formation dramatically reduced the level of infection (Mazurov et al., 2010).

Subsequent studies have shown that the MTOC also polarizes in HIV-1-infected cells during VS formation, and that the polarization of the viral proteins involves actin and tubulin (Jolly et al., 2004, 2007; Chen et al., 2007; Sol-Foulon et al., 2007; Vasiliver-Shamis et al., 2009). For HIV, it has recently been proposed that the delivery of viral proteins following the polarization of the infected cell involves the regulated secretory pathway of CD4^+^ T cells (Jolly et al., 2011). It was observed that the polarization of the MTOC is associated with the alignment of organelles involved in the secretory pathway, that these organelles colocalize with HIV Env proteins, and that cells with a genetic defect in this pathway were less efficient at cell–cell transmission of the virus (Jolly et al., 2011). Because of the other similarities between the HTLV-1 and HIV VS, it seems possible that HTLV-1 could also hijack this secretory pathway to facilitate cell–cell transmission.

Little is known about the fate of HTLV-1 following transmission across the VS. However, for HIV, a number of recent studies have provided insight into the steps required for productive infection following transmission via the VS. Several recent studies support a model that involves two steps prior to productive infection of CD4^+^ T cells. It has recently been observed that, following budding from the polarized infected cell, immature HIV viral particles enter an internal endocytic compartment of the target cells, a step that has been called cell-to-cell transfer (Bosch et al., 2008; Hubner et al., 2009; Puigdomenech et al., 2009). The virus then matures, which allows Env-mediated fusion of the viral and cellular membranes in the endocytic compartment (Dale et al., 2011). Future work is needed to investigate whether a similar pathway is used by HTLV-1 following transmission via the VS.

For HTLV-1, the virus-encoded transactivating protein Tax has been shown to contribute to VS formation and cytoskeletal polarization in several related ways. Tax upregulates expression of ICAM-1 (Tanaka et al., 1995) which, as described above, facilitates VS formation by interacting with LFA-1 on the uninfected cells (Barnard et al., 2005). Tax has been observed to localize to a region near the point of contact, and there is some evidence that Tax triggers MTOC polarization by enhancing signaling through ICAM-1, and possibly other molecules (Barnard et al., 2005; Giam and Jeang, 2007; Nejmeddine et al., 2009). The importance of Tax during cell–cell transmission was confirmed in infectivity assays with HTLV-based vectors: in the absence of Tax, infectivity was reduced more than a log (Mazurov et al., 2010). This effect was dependent on the infected cell type (Tax enhanced cell–cell spread from T cells but not from fibroblasts) and the Env present on the HTLV-1 core (the infection levels of VSV-G pseudotyped virions were not increased in the presence of Tax) (Mazurov et al., 2010). Thus, it appears that Tax plays a role in enhancing interactions between HTLV-1 Env and adhesion molecules as well as by facilitating the movement of Env and Gag to the VS.

The HTLV-1 protein p8, which is produced upon cleavage of the auxiliary protein p12, has also been reported to enhance interactions between HTLV-1-infected cells and uninfected cells. The p8 protein also promotes cell–cell transmission of the virus by increasing ICAM-1/LFA-1 interactions (Van Prooyen et al., 2010). This protein colocalizes with and increases clustering of LFA-1 on the surface of the infected T cell, which enhances T cell interactions by facilitating interactions of LFA-1 with ICAM-1 (Van Prooyen et al., 2010).

## TRANSMISSION BY FILOPODIA, NANOTUBES, OR CONDUITS

Previous studies have shown that HIV and MLV can also be rapidly transferred between infected and uninfected cells via transient membrane tethers including filopodia and nanotubes (Sherer et al., 2007; Sherer and Mothes, 2008; Sowinski et al., 2008; Nobile et al., 2009). Viral particles have been observed on the outer surface of the filopodia, finger-like membrane protrusions from cells, that formed a bridge between the virus-producing cells and the target cells (Sherer et al., 2007). For nanotubes, which form *de novo* between immune cells separating following contact (Onfelt et al., 2004), viral particles have been observed within the nanotubes, transferring between the cells (Sowinski et al., 2008; Eugenin et al., 2009). For both these routes of infection, as for the VS, entry of the virus into the target cell requires interactions between the viral Env proteins and the receptors on the uninfected cell (Sherer and Mothes, 2008; Sowinski et al., 2008).

Recently it has been reported that HTLV-1 can also spread from an infected to an uninfected T cell by membrane extensions, which the authors refer to as cellular conduits (Van Prooyen et al., 2010). Transmission EM studies revealed that, as occurs during formation of the VS, the HTLV-1 particles are concentrated at the point of contact between the HTLV-1-infected cell and the target cell, either between conduits (**Figure 1B**) or between conduits and cells (not shown). In addition to its role in enhancing LFA-1/ICAM-1 interactions, overexpression of the accessory protein p8 in HTLV-1-infected cells was observed to increase the number and length of these conduits as well as the number of contacts between infected and uninfected cells (Van Prooyen et al., 2010). The HIV-1 accessory protein Nef was also shown to induce the formation of nanotubes or conduits (Xu et al., 2009; Mukerji et al., 2012). However, in contrast to p8, Nef does not appear to significantly modulate the formation of the VS (Haller et al., 2011).

## TRANSMISSION VIA ADHESIVE VIRAL ASSEMBLIES

Transmission of HTLV-1 and other viruses through the VS and the other cell–cell connections described above involve membrane-to-membrane contact, either between two cells in close contact (in the case of VS) or at a distance (for filopodia and conduits). Recently it has been reported that retroviruses can also be transmitted from cell-to-cell via clusters of particles attached at the surface of the producing cells.

HTLV-1-infected CD4^+^ T cells have been observed to have clusters of mature viral particles on their surface (Pais-Correia et al., 2009). These virions are present within a specific type of matrix synthesized by the producing cells, which the authors named a biofilm, that link the virions to the cell surface as well as to each other (**Figure 1C**). This viral/cellular network is enriched in specific components of the extracellular matrix (ECM), including HSPGs, agrin, collagen, and the glycan-binding protein galectin-3. When CD4^+^ T cells from HTLV-1-infected individuals were exposed to uninfected CD4^+^ T cells, these assemblies were quickly transferred to the target cell (Pais-Correia et al., 2009). The ECM components were observed to colocalize with viral proteins, indicating that the HTLV-1 was being transferred in the context of the viral assemblies. Infectivity studies validated the indication from these imaging studies that these virus assemblies enhanced cell–cell transmission of the virus: removing these viral assemblies from the surface of the infected cells, either mechanically or by treatment with heparin, reduced the number of newly infected target cells by more than 80% (Pais-Correia et al., 2009).

Studies of MLV-infected fibroblast cell lines have also shown that virus assemblies on the cell surface can enhance cell–cell transmission. Newly budded viruses were observed to remain associated with the surface of infected cells and removing heparan sulfate from the cell surface caused the viruses to detach (Sherer et al., 2010). As was observed for HTLV-1, MLV particles on the surface of infected cells are transmitted following contact with uninfected cells.

Reports that cell–cell transmission of HTLV-1 occurs by VS and by transfer of extracellular viral assemblies, in studies using similar techniques on similar cells, raises the question of whether the different laboratories are observing the same phenomena but reaching different conclusions, or whether they were truly observing distinct phenomena. Moreover, as described above, one group observed evidence of HTLV-1 transmission via both membrane extensions and VS in the same cocultures of infected and uninfected cells (Van Prooyen et al., 2010). It is important to note that, as elegantly argued in a comment on the study describing the HTLV extracellular viral assemblies (Jin et al., 2010), different methods of transmission are not mutually exclusive. It is possible that HTLV-1 spreads both by surface transmission during transient contacts and by polarized assembly and transfer following longer cell–cell interactions. The authors' inclusive view about HTLV-1 transmission is likely informed by their laboratory's own observations that MLV can be transmitted following polarized viral assembly and longer term cell–cell interactions via a VS (Jin et al., 2009) and by longer membrane extensions (filopodial bridges; Sherer et al., 2007; Sherer et al., 2010), as well as by the transfer of cell-surface bound virus during transient interactions (Sherer et al., 2010).

## HTLV-1 TRANSMISSION BY ANTIGEN-PRESENTING CELLS

In addition to spreading between T cells, HTLV-1 can be transmitted from DCs to CD4^+^ T cells. *In vitro* studies have shown that DC-mediated HTLV-1 infection of T cells can occur in two different ways. In one mode of transmission (*cis*-infection), the DCs are infected, and then the *de novo* produced HTLV-1 is transferred to the T cells (Jones et al., 2008). In the other (*trans*-infection), uninfected DCs capture virus produced by infected T cells, and transmit the virus to T cells prior to becoming infected themselves (**Figure 1D**; Jones et al., 2008; Jain et al., 2009).

Both *in vivo* and *in vitro* studies support the notion that transmission to T cells via DCs plays a role in the spread of HTLV in infected individuals. The two major types of DCs in peripheral blood and lymphoid tissues are plasmacytoid DC (pDCs) and myeloid DCs (myDCs). Studies examining pDCs and myDCs isolated from the peripheral blood of infected individuals have shown that they are infected with HTLV-1 (Macatonia et al., 1992; Knight et al., 1993; Hishizawa et al., 2004). Moreover, pDCs isolated from HTLV-1-infected individuals and cultured *ex vivo* have been shown to be able to transmit virus to, and productively infect, CD4^+^ T cells (Jones et al., 2008). In addition, primary pDCs and myDCs isolated from the peripheral blood of uninfected individuals can be infected by cell-free virus *in vitro*, and these cells can infect autologous primary CD4^+^ T cells by the *de novo* produced virus (Jones et al., 2008). *In vitro* generated monocyte-derived DCs (MDDCs) can also be infected by cell-free HTLV-1, and by cell–cell interactions with infected CD4^+^ T cells (Ceccaldi et al., 2006; Jones et al., 2008; Jain et al., 2009; Lambert et al., 2009; Valeri et al., 2010; Martin-Latil et al., 2012).

As mentioned above, DC-mediated infection can also occur *in trans*, by DCs that bind HTLV-1 and transfer the virus to CD4^+^ T cells prior to becoming infected themselves. This has been shown for pDCs and myDCs as well as MDDCs (Jones et al., 2008; Jain et al., 2009). One protein that has been identified as important for efficient transfer of HTLV-1 *in trans* from MDDCs to T cells**is DC-specific ICAM-3-grabbing non-integrin (DC-SIGN; Jain et al., 2009), a lectin previously shown to mediate transfer of HIV during *in trans* infection of CD4^+^ T cells via MDDC. DC-SIGN expression on uninfected DC has also been shown to promote transmission of HTLV-1 from T to DC cells, by enhancing interactions with ICAMs on infected CD4^+^ T cells (Ceccaldi et al., 2006). As mentioned above, on the target CD4^+^ T cell, efficient *in trans* transmission of HTLV-1 from infected DCs requires both NRP-1 and HSPG (Jones et al., 2008).

The cell–cell interactions that occur during transmission of HTLV-1 from DC to T cells, either when transmitted *in trans *or from infected cells, have not been well characterized. To date, no imaging studies examining interactions during the DC-mediated infection of T cells by HTLV have been published. HIV, like HTLV-1, can be transmitted to CD4^+^ T cells via DCs either *in trans* following the capture of virus by uninfected DCs (McDonald et al., 2003) or *in cis* during cell–cell transmission from infected DCs to uninfected CD4^+^ T cells (Burleigh et al., 2006). For the *trans*-infection of HIV via MDDCs, early imaging studies showed that, similar to what occurs during T cell–T cell transmission, viral particles are concentrated on DC at the site of contact, CD4 and chemokine co-receptors are recruited to the contact site on the T cell surface, and virions are transferred to T cells (McDonald et al., 2003; Arrighi et al., 2004; Turville et al., 2004; Garcia et al., 2005; Wang et al., 2007). More recent studies using super resolution light microscopy, ion abrasion scanning electron microscopy, and electron tomography have revealed that HIV is transferred from surface-accessible compartments within the DCs to the T cells, and that these interactions can involve membrane extensions of the DCs and T cells (Yu et al., 2008; Felts et al., 2010; Nikolic et al., 2011). Less is known about interactions between infected DC and CD4^+^ T cells during the transfer of *de novo* produced HIV. One recent study of infected MDDCs observed the presence of HIV particles on the tips of the majority of the filopodia. Using real-time imaging, these viral-containing filopodia were observed to contact and tether to the CD4^+^ T cells, and then reposition and converge to become the DC-T cell viral synapse (Aggarwal et al., 2012).

Several lines of evidence suggest that cells of the monocyte/macrophage lineage may also be involved in cell–cell transmission of HTLV-1. Like DC, cells of this lineage isolated from HTLV-1-infected individuals have been reported to be infected with the virus (Koyanagi et al., 1993) and primary cultures of monocytes, macrophages, and microglial can be productively infected *in vitro* following exposure to cell-free HTLV-1 (Hoffman et al., 1992; Koralnik et al., 1992). Macrophages isolated from breast milk as well as from peripheral blood can also be infected *in vitro *by coculture with HTLV-1-infected T cells (de Revel et al., 1993; Takeuchi et al., 2010). Moreover, breast milk macrophages can be persistently infected and transformed with HTLV-1, and these cells can transmit virus to activated T cells (Takeuchi et al., 2010).

These observations raise the possibility that macrophages play a role in the persistence of HTLV-1. This has been well documented for HIV: macrophages can be productively infected with HIV (Gendelman et al., 1988), and HIV-infected macrophages are believed to be an important part of the viral reservoir (reviewed in Le Douce et al., 2010). In contrast to T cells, where the virus is assembled and released from the plasma membrane, infected macrophages accumulate HIV in large virus-containing compartment (VCC) inside the cells (Raposo et al., 2002; Nydegger et al., 2003; Pelchen-Matthews et al., 2003; Sherer et al., 2003; Ono and Freed, 2004; Deneka et al., 2007). Imaging studies have shown that HIV released from the VCC can be transmitted to CD4^+^ T cells via a VS (Gousset et al., 2008; Groot et al., 2008; Bennett et al., 2009). Further studies are needed to determine whether macrophages play a similar role in the infection and persistence of HTLV-1.

## CELL–CELL INTERACTIONS DURING HTLV-1 TRANSMISSION BETWEEN INDIVIDUALS

HTLV-1 is transmitted in three ways: from mother to infant, by sexual contact, and through HTLV-1-infected blood or cellular blood products (reviewed in Goncalves et al., 2010). This has been assumed to involve the transfer of HTLV-1-containing T cells in the bodily fluids of the infected individuals followed by interactions with, and infection of, T cells in the recipient. However, the specific cell types from either the infected or the uninfected individuals involved in this transmission have not yet been characterized. In addition to T cells, a significant percentage of pDCs and myDCs in peripheral blood are infected with HTLV-1 in some individuals (Hishizawa et al., 2004; Azakami et al., 2009). One study reported that the HTLV-1 proviral load in pDCs was higher than in PBMCs from the same individual (Hishizawa et al., 2004). Along with studies demonstrating that HTLV-1 is efficiently transmitted to CD4^+^ T cells via DCs *in vitro* (Jones et al., 2008) and recent insights into the cells involved in transmission of HIV and simian immunodeficiency virus (SIV) described below, these observations support the notion that cells other than T cells are involved in HTLV-1 transmission between individuals.

## MOTHER-TO-CHILD TRANSMISSION

In endemic areas, the primary route of transmission of HTLV-1 is from infected mother to child. The rate of vertical transmission in endemic populations has been estimated to be between 10 and 25% (Tsuji et al., 1990; Hirata et al., 1992; Nyambi et al., 1996; Takezaki et al., 1997; Wiktor et al., 1997; Biggar et al., 2006). In the vast majority of these cases, HTLV-1 infection occurs by ingestion of breast milk: mother-to-child transmission occurs in less than 5% of women who do not breastfeed their children, indicating that transplacental and perinatal infection are uncommon (Kinoshita et al., 1987; Caterino-de-Araujo and de los Santos-Fortuna, 1999; Kashiwagi et al., 2004). Moreover, the risk of HTLV-1 infection increases with the duration of breastfeeding (Hirata et al., 1992; Takezaki et al., 1997; Wiktor et al., 1997) and public health policies in Japan encouraging infected women to avoid breastfeeding have dramatically reduced the number of mother-to-child infections (Kashiwagi et al., 2004)*. *Vertical transmission of HTLV-2 also occurs in this manner: two independent studies saw no evidence of mother-to-child transmission in women who did not breastfeed their children (Kaplan et al., 1992; Gallo et al., 1993).

Several lines of evidence indicate that HTLV-1 transmission via breast milk, like transmission from blood products, involves infected cells. Cells isolated from the breast milk have been shown to contain HTLV-1 proviral DNA and epidemiological studies have shown that the risk of infection in children correlates with the provirus load in these cells (Li et al., 2004). The fact that cells are sufficient for viral transmission has been shown in an animal model system:**HTLV-1 was transmitted to non-human primates following oral infection with cells isolated from the breast milk of HTLV-1-infected women (Kinoshita et al., 1985).**No studies performed to date have examined whether cell-free HTLV-1 virions are present in the breast milk of infected women.

The majority of HTLV-1 infections occur in children who have been breastfed for more than 6 months (Takezaki et al., 1997). At that time, the majority of the cells in breast milk (>80%) are T cells; monocytes, macrophages, B cells, and epithelial cells are also present (Southern and Southern, 1998; Kelly and Coutts, 2000). Studies with *ex vivo* cultures of luminal epithelial cells isolated from the milk of uninfected women revealed that these epithelial cells can become infected following exposure to HTLV-1-infected T cells. The HTLV-1-infected epithelial cells can become transformed, and these transformed cells spontaneously produce an ECM (Southern and Southern, 1998). Moreover, both T lymphocytes isolated from peripheral blood and epithelial cells isolated from the gut were infected following coculture with these transformed, HTLV-1-infected cells (LeVasseur et al., 1998; Southern and Southern, 1998). In light of the more recent studies characterizing the extracellular viral assemblies described above, it is interesting to note that the level of infection in this study was reported to be higher for the T cells cultured with HTLV-1-infected epithelial cells attached to the ECM than those cultured with non-adherent aggregates of the same cells.

In addition to T cells and epithelial cells, breast milk macrophages also may play a role in transmission: it has recently been reported that these cells can be infected with HTLV-1 and that a cell line generated from HTLV-1-infected breast milk macrophages can transmit virus to primary T cells (Takeuchi et al., 2010)*.*

**FIGURE 2:**
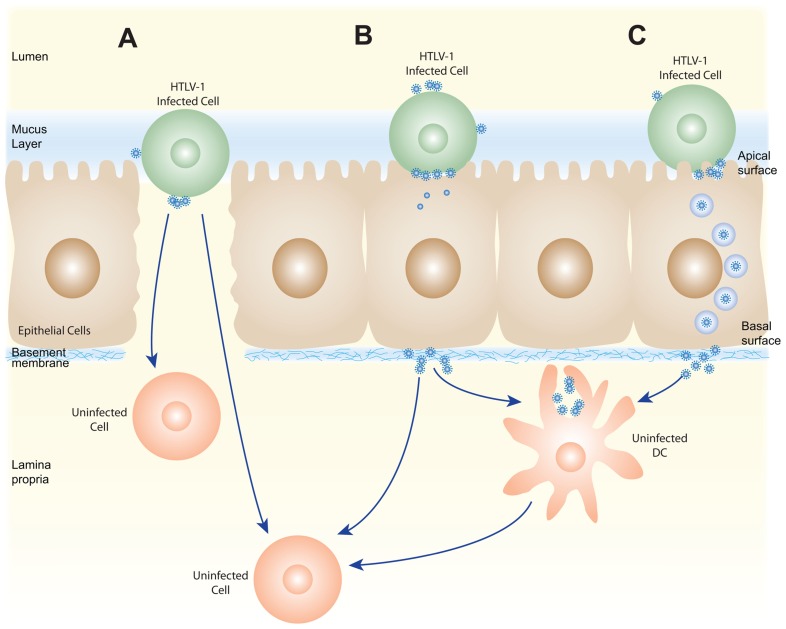
**Schematic of several possible pathways for HTLV-1 transmission via breast milk**. HTLV-1-infected cells present in breast milk (T cells, macrophages, and/or epithelial cells) could transmit the virus across the oral or gastrointestinal mucosa in several ways. The HTLV-1-infected cells could cross the barrier at positions where the mucosa are disrupted **(A)** or by transmigration across the epithelial barrier (not shown). HTLV-1 could then be passed by cell–cell transmission to the target cells present in the lamina propia (T cells, macrophages, DC/Langerhans) or in the draining lymph nodes. Alternatively, the HTLV-1-infected cells could infect epithelial cells by cell–cell transmission of the virus **(B)**, and these productively infected epithelial cells could either directly infect target cells in the lamina propia, or produce virus that is captured by a DC or other APC. Following capture, HTLV-1 could be transmitted to susceptible cells either in the lamina propia or lymph nodes. The virus produced by HTLV-1-infected cells could also by transmitted without infecting epithelial cells by transcytosis **(C)**, be captured by APC, and subsequently used to infect target cells.

After the HTLV-1-infected cells enter the digestive tract, infection would likely involve transfer of HTLV-1-infected cells and/or cell-free HTLV-1 produced by those infected cells across the epithelium in the oral or gastrointestinal mucosa. This could occur in a number of different ways, some of which are shown in **Figure 2**. HTLV-1-infected cells could be transmitted at places where the integrity of mucosa is disrupted or where there are gaps in the epithelium (**Figure 2A**). It is also possible that HTLV-1-infected cells could cross intact epithelium: HIV-infected macrophages have recently been shown to be able to transmigrate across fetal oral epithelia (Tugizov et al., 2012). Alternatively, the HTLV-1-infected cells could attach to the apical side of the epithelial cells, infect the cells by cell–cell transmission of the virus, and the newly produced viral particles could bud from the basal surface (**Figure 2B**). This model is supported by *in* *vitro* studies showing that HTLV-1-infected T cells can adhere to the microvilli of a monolayer formed by an intestine-derived epithelial cell line, that a large number of viral particles are released into the space between the cells, and that the epithelial cells became infected (Zacharopoulos et al., 1992). Another way that the cell-free HTLV-1 virions produced by infected cells could be transmitted through an intact epithelial barrier is by being taken up by a vesicle on the apical surface of an epithelial cell and transported to and released from the basal side (**Figure 2C**). HTLV-1 has recently been shown to be capable of crossing an epithelial barrier by this process, which is referred to as transcytosis: virus produced from infected T cells was transported across a tight epithelial barrier in an *in vitro *model of human intestinal cells (Martin-Latil et al., 2012). Moreover, the transmitted virus was able to infect human DCs located immediately below the epithelial barrier (Martin-Latil et al., 2012)*. *HIV-1 has also been shown to be capable of crossing intact oral and intestinal epithelial barriers by transcytosis and to be capable of infecting DCs, macrophages, and CD4^+^ T cells on the other side of those barriers in both *ex vivo* studies using tissue explants and *in vitro* studies (Bomsel, 1997; Alfsen et al., 2005; Tugizov et al., 2012).

In the model of infection just described, the cells in the child would become infected after the HTLV-1 crossed the oral or gastrointestinal mucosa, either as an infected cell or as a cell-free particle. It is possible that HTLV-1-infected cells, either T cells or antigen-presenting cells (APCs), could then infect resident T cells immediately after the barrier was crossed. T cells, DCs, and macrophages have all been observed to be present in the oral mucosa of infants (Tugizov et al., 2012).

If cell-free virus crossed the barrier, DC or other APCs could either become infected or capture HTLV-1. These APCs could transfer the virus to either resident T cells or T cells present in secondary lymphoid organs after exiting the mucosa and migration. It is interesting to note that the basal surface of the epithelial cells is connected to the basement membrane, which contains components of the ECM present in the extracellular viral assemblies. It is possible that these could interact with the cell-free particles to enhance transmission to the target cells.

## SEXUAL TRANSMISSION

The other natural route of infection of HTLV-1 is by sexual contact. Although early studies suggested that male-to-female transmission of HTLV-I infection was much more frequent than female-to-male transmission, later prospective studies have shown that this difference is not as dramatic as previously believed (Roucoux et al., 2005). As is the case for transmission from mother to child, little is known about what infected cells are transmitted from men or women with HTLV-1, or what cells are the initial targets in the previously uninfected individual. Although no studies performed to date have examined which cells are infected in the semen of HTLV-1-infected men, semen in healthy men contain several different cell types including CD4^+^ T cells and macrophages (reviewed in Anderson et al., 2010), suggesting that one or both of these cells are the source of the virus. In regard to female-to-male transmission, HTLV-1-infected cells have been detected in cervical secretions of infected women (Belec et al., 1996; Zunt et al., 2002), although the infected cell types were not identified. CD4^+^ T cells can be detected in cervicovaginal secretions from healthy women, although the numbers are very low. However, the number of CD4^+^ T cells is increased in cervicovaginal secretions with certain infections; this is consistent with the observation that detection of HTLV-1-infected cells in cervical secretions was associated with inflammation of the uterine cervix (Zunt et al., 2002).

Sexual transmission of HTLV-1 would presumably require entry through mucosal barriers in the female and male genital tracts. As during transmission by breastfeeding, the virus could be transmitted where lesions disrupt the mucosa, by infection of the epithelium or by transcytosis across epithelial cells. Female-to-male transmission is higher in men with syphilis or a history of penile sores or ulcers (Murphy et al., 1989), consistent with the notion that disrupting the epithelium increases transmission. Infection of a cervix-derived epithelial line following exposure to HTLV-1-infected T cells has been shown *in vitro *(Zacharopoulos et al., 1992), and HTLV-1 has been associated with carcinoma of the cervix (Strickler et al., 1995), suggesting that viral infection of epithelium or other cells in the cervix may play a role in male-to-female spread.

Although the mechanism of this transmission has not been examined for HTLV, mucosal entry of HIV and SIV in the genital tracts have been well characterized by *in vitro* studies of purified cell populations, *ex vivo* studies using human explants, and *in vivo* studies in macaques and humanized mice (reviewed in Hladik and McElrath, 2008; Ganor and Bomsel, 2011; Kaushic, 2011). These studies have shown that HIV can be transmitted across the epithelium by all of the methods described above: infected seminal cells, or virus produced by those cells, have been shown to cross through gaps in the epithelium, by infecting the epithelium, or by transcytosis.

Once viruses have crossed the mucosal barriers, as with infection via breast milk, they can infect DCs, macrophages, or T cells. For HIV, a number of studies suggest that APCs capture the viruses and transfer them to T cells. For example, study performed with a model for HIV-1 female-to-male transmission using foreskin explants observed that virus-infected cells form a VS with the apical side of foreskin keratinocytes and that HIV-1 subsequently budding from the basal side is captured by Langerhan cells (LC), a type of DC. LC then migrate to the epidermis–dermis interface and transfer the virus to T cells (Ganor et al., 2010)*. Ex vivo* human organ culture systems for male-to-female transmission have shown that intraepithelial vaginal LCs can capture HIV and that these cells can productively infect T cells (Hladik et al., 2007; Ballweber et al., 2011).

## TRANSMISSION BY BLOOD

As discussed in the introduction, HTLV-1 can be transmitted by transfusion of whole blood or blood products containing cells. Transmission by blood also occurs during the sharing of needles by intravenous drug users. Unlike the natural methods of infection described above, transmission via contaminated blood does not require transmission across a mucosal barrier. Consistent with that notion, transmission via blood is efficient: one prospective study showed seroconversion of 44% of recipients after a single exposure to HTLV-1-infected cellular blood products (Manns et al., 1992). Because of the lack of a barrier, this method of transmission could involve direct transmission of HTLV-1 from the cells of the infected individual to target cells in the previously uninfected individual. Since the virus is predominantly found in T cells in peripheral blood, this type of transmission could occur during direct interactions between T cells by the methods that have been described for *in vitro* T cell–T cell transmission: via VS, by transfer of extracellular viral assemblies, or other methods. Since myDCs and pDCs from peripheral blood of asymptomatic individuals are infected with HTLV-1 (Hishizawa et al., 2004; Azakami et al., 2009) transmission from DC to T cells may also occur.

Individuals who acquire HTLV-1 by blood transfusion are more likely to develop the chronic inflammatory disorder HAM/TSP (Osame et al., 1990), while individuals who acquire the virus during breast feeding are more likely to develop the T cell malignancy ATL (Kakuda et al., 2002). While this could reflect a number of other factors (including age of infection, amount of virus acquired during transmission, and immune response), it has been suggested that these different methods of infection result in different populations of infected cells, which in turn influences which disease a given infected individual is more likely to develop.

## ESTABLISHMENT OF HTLV-1 INFECTION

As described in the introduction, it is believed that infection of an individual with HTLV-1 occurs in two stages. The virus is thought to initially spread from T cell to T cell, primarily between CD4^+^ T cells, and later to persist by clonal expansion of infected cells. Although nearly all studies of HTLV-1 performed to date have focused on studies of T cells from peripheral blood, it would seem likely that at least some of this spread would occur in the lymph nodes, where the concentration of the T cells is higher and the T cells are highly motile. The notion that a significant number of HTLV-1-infected cells are present outside the peripheral blood is supported by observations of a strong immune response (HTLV-1-specific T cells and antibodies) in chronically infected individuals, even when no virus or virus-expressing cells can be detected in the peripheral blood.

One recent study of HIV transmission provides a model for how the observations of cell–cell transmission via VS observed for HTLV-1 and HIV-1 *in vitro* might be relevant to *in vivo* transmission in lymph nodes (Llewellyn et al., 2010). In lymphoid organs, such as lymph nodes, T cells are highly motile and adopt an elongated or polarized morphology. When HIV-1-infected polarized T cells were examined, Gag was found to localize to a rear end protrusion known to mediate contact with other cells called a uropod. The uropods preferentially form contacts with target cells, and these uropod-mediated contacts eventually form a VS (Llewellyn et al., 2010). It seems possible that, soon after an individual is infected with HTLV-1, the virus spreads between T cells in the lymph nodes using a similar mechanism.

It is also possible that DCs or other APC cells contribute to the cell–cell spread during this early stage of transmission. DCs can be highly mobile and, as described above, are infected at a significant level in many individuals with HTLV-1. This notion that virus may be passed from T cells to DC and then back to T cells is also supported by the *in vitro* observations that both DCs can be infected by HTLV-1-infected T cells and T cells can be infected by HTLV-1-infected DC.

Another possible reservoir for HTLV-1 is human CD34^+^ hematopoietic progenitor/stem cells (HP/HSCs). HTLV-1 can infect these cells when cultured *ex vivo*, and the proviral genome is maintained when the cells are differentiated down several lineages, including T cells and monocyte/macrophage lineages (Feuer et al., 1996). Moreover, a significant number of SCID mice in which hematopoiesis is reconstituted with HTLV-1-infected CD34^+^ HP/HSCs develop lymphoma similar to ATL (Banerjee et al., 2010). CD34^+^ HP/HSCs isolated from infected individuals have been reported to contain HTLV-1 sequences (Banerjee et al., 2010), and, in one case, transmission of HTLV-1 occurred following allogeneic BM transplantation (Kikuchi et al., 2000).

## CONCLUSIONS AND PERSPECTIVES

Insights gained in the last 10 years have dramatically changed our understanding about how HTLV is transmitted between cells. The long held beliefs that efficient transmission of HTLV-1 and HTLV-2 requires interactions between an infected and an uninfected T cell, and that this mode of transmission distinguishes these viruses from other retroviruses, no longer appears to be the case. While a decade ago little was known about the interactions between cells required for HTLV-1 transmission, recent imaging studies have identified some of the components of the infected cell, the uninfected cell, and the virus that facilitate different types of cell–cell transmission. The more detailed studies of HIV-1 cell–cell transmission and other retroviruses have provided additional clues about how HTLV-1 may be transmitted. These studies, along with *in vitro* studies of infectivity, have revealed that T cells can be infected by cell–cell transmission from infected non-T cells as well as T cells. They have also demonstrated that cell–cell transmission of HTLV-1 and HIV to a target cell can occur via an uninfected APC that has captured viral particles, as well as from an infected cell.

These studies suggest that the mechanism of transmission between individuals, and within newly infected individuals prior to the clonal expansion that characterizes chronic infection by HTLV-1, differs from the current dogma that cell–cell transmission of HTLV-1 only involves interactions between T cells. Observations that macrophages and epithelial cells isolated from breast milk can be infected and transformed with HTLV-1 suggest that transmission via breast milk might involve these cells as well as T cells. The fact that HTLV-1-infected DCs are present in the peripheral blood of infected individuals, and that T cells are efficiently infected by DC *in vitro,* suggests that DCs or other APC play a role in HTLV-1 transmission between individuals, and/or during the initial spread of infection in an individual. This is supported by the large number of studies indicating that DC and other APC play a role during mucosal entry of HIV and SIV.

Despite our recent advances, there are many aspects of HTLV-1 transmission that remain poorly understood. Nothing is known about whether productive infection following cell–cell transmission of HTLV-1 occurs following endocytosis of the virus by the target cell and/or by fusion at the cellular surface. Since cell-free HTLV has been shown to efficiently bind and enter primary T cells, it is unclear whether the block to stable infection reflects uptake by a non-productive pathway, restriction by intracellular antiviral factors, a negative effect of viral expression in the cells, or other factors. Although one can speculate (as we have in the previous section) about what cells are involved during transmission between individuals by mucosal and parenteral routes based on studies of lentiviral transmission, only a few, limited studies have been performed on fluids from HTLV-1-infected individuals or with *ex vivo* cultured cells and tissues to address this directly. In addition, once an individual is infected with the virus, it is unclear where, or between what cell types, the virus initially spreads prior to clonal expansion of the infected T cells, and whether HTLV-1 persists in reservoirs outside the peripheral blood during the chronic stage of infection. Future studies investigating these and other aspects of HTLV transmission and persistence will be important for developing therapeutics to block initial infection with the virus, as well as to reduce the level of cells containing the virus in infected individuals.

## Conflict of Interest Statement

The authors declare that the research was conducted in the absence of any commercial or financial relationships that could be construed as a potential conflict of interest.
